# Histological, ultrastructural, and single-cell profiling reveal immune-mediated remodeling in gallbladder inflammation

**DOI:** 10.1007/s00441-026-04057-6

**Published:** 2026-03-05

**Authors:** Dmytro Vlasenko, Andrea Maccagno, Adriano Sanna, Tamara Papadakis, Shanjid Ahmed Shiplu, Ramona Schmid, Ulrich Gärtner, Bruno Märkl, Marco Koch, Maryam Keshavarz

**Affiliations:** 1 Department of General, Visceral and Transplantation Surgery, University Hospital Augsburg, Augsburg, Germany; 2 Institute of Pathology and Molecular Diagnostics, Faculty of Medicine, University of Augsburg, Augsburg, Germany; 3 Institute for Anatomy and Cell Biology, German Center for Lung Research, Justus Liebig University, Giessen, Germany; 4 Anatomy and Cell Biology, Institute of Theoretical Medicine, Faculty of Medicine, University of Augsburg, Augsburg, Germany; 5 Dean’s Office, Faculty of Medicine, University of Augsburg, Augsburg, Germany; 6 Centre for Interdisciplinary Health Research (CIHR), University of Augsburg, Augsburg, Germany; 7 Centre for Advanced Analytics and Predictive Sciences (CAAPS), University of Augsburg, Augsburg, Germany

**Keywords:** Gallbladder, Cholecystitis, Inflammation, Epithelium, Macrophage, Fibrosis

## Abstract

Gallbladder inflammation comprises distinct pathological entities, including acute, neutrophil-dominated injury and chronic, fibrotic remodeling. This study aimed to define the cellular and structural programs that characterize these two inflammatory states and link epithelial, immune, and stromal alterations. Tissue and blood samples from forty-one patients, including twelve with acute cholecystitis and twenty-nine with chronic cholelithiasis, were analyzed using histopathology, immunohistochemistry, transmission electron microscopy, targeted cytokine expression analysis, and single-cell RNA sequencing of immune-enriched suspensions. Acute cholecystitis showed epithelial disruption, edema, and dense infiltration by neutrophils and macrophages, including an increased density of CD163^+^ macrophages, accompanied by elevated systemic inflammation. Chronic cholelithiasis displayed preserved epithelial continuity, fibrosis, glandular remodeling, and reduced immune-cell density. Ultrastructural analysis revealed abundant mucin granules and intact junctions in acute inflammation, contrasting with mucin depletion and dense-body accumulation in chronic disease. Single-cell transcriptomic analysis identified twelve immune and stromal populations, showing contrasting immune–stromal configurations: pro-inflammatory myeloid and cytotoxic T cells dominated in acute inflammation, whereas macrophage–B-cell–fibroblast networks were enriched in chronic cholelithiasis, reflecting adaptive and fibrotic remodeling rather than a temporal transition. This study defines distinct but coordinated immune–stromal programs underlying human gallbladder inflammation and provides a cellular framework for understanding condition-specific mechanisms of acute and chronic disease.

## Introduction

Gallbladder inflammation is a common and clinically significant disorder of the biliary system, most often associated with gallstone formation and ranging from mild inflammation to severe septic or perforated forms (Costanzo et al. 2023). Despite its high prevalence, the cellular and regional mechanisms underlying epithelial injury and immune activation remain incompletely understood (Gutt et al. 2020; Kratzer et al. 1998; Lam et al. 2021; Shabanzadeh 2018). Management strategies for gallstone disease remain debated, particularly regarding the indication for prophylactic or simultaneous cholecystectomy in asymptomatic patients, for whom the clinical benefit remains uncertain (Indar and Beckingham 2002; Lam et al. 2021). Current guidelines recommend surgery only in the presence of biliary symptoms, emphasizing the need to better understand the mechanisms that drive symptomatic inflammation.

Two major entities define the clinical and histopathological spectrum of gallbladder inflammation: acute cholecystitis and chronic cholelithiasis. Acute cholecystitis results from cystic-duct obstruction, leading to bile stasis, ischemic epithelial injury, and transmural infiltration by neutrophils (Yokoe et al. 2018). Chronic cholelithiasis evolves through persistent mechanical irritation and low-grade inflammation, culminating in smooth-muscle hypertrophy, wall thickening, and fibrosis (Jones et al. 2025; Lammert and Wittenburg 2024). Although the histological distinctions between acute cholecystitis and chronic cholelithiasis are well established, the cellular mechanisms and immune–epithelial signaling pathways distinguishing these inflammatory states remain poorly defined. Cystic-duct obstruction initiates bile stasis and distension, but the ensuing inflammation also involves epithelial secretion of mucins, prostaglandins, and microbial factors that amplify immune activation (Fukunaga 1973; Roslyn et al. 1980; Thornell et al. 1986).

The gallbladder epithelium is a highly specialized single-layered mucosa composed of absorptive and secretory columnar cells responsible for bile concentration and maintenance of bile composition. Its function depends on the coordinated transport of ions, water, and mucins, and disruption of these processes can induce bile stasis, alter bile rheology, and trigger inflammation (Portincasa et al. 2006). Overexpression of mucins such as MUC2 and MUC5B promotes mucus accumulation and gallstone nucleation (Debray et al. 2012; Deng et al. 2021; Gookin et al. 2024). Moreover, biliary epithelial cells secrete inflammatory mediators including TNF-α and IL-6, reflecting an active immunoregulatory role during gallbladder inflammation (Kasprzak et al. 2015; O'Leary et al. 2022).

Importantly, the regional architecture of the human gallbladder is highly heterogeneous. The neck region, continuous with the cystic duct, contains dense glandular invaginations and a rich immune and neural network, whereas the body exhibits a thinner mucosa specialized for fluid absorption (Prozorowska and Jackowiak 2015). The peribiliary glands, located in the neck and proximal cystic duct, act as epithelial progenitor niches that secrete both mucins and antimicrobial peptides (Han et al. 2013; Lan et al. 2022; Nakanuma et al. 1997). These glands are critical for epithelial regeneration and defense, particularly following inflammation or biliary obstruction. However, how these distinct epithelial compartments respond differentially during acute versus chronic inflammation, and how they coordinate with immune-cell infiltration, remains poorly understood.

Acute and chronic cholecystitis exhibit distinct structural alterations from epithelial atrophy and polarity loss in acute inflammation to fibrosis and mucin accumulation in chronic disease (Gallaher and Charles 2022a, 2022b; Giurgiu et al. 1997). Yet, how these morphological states emerge from dynamic immune–epithelial interactions remains unresolved. By integrating histopathological, ultrastructural, and single-cell transcriptomic analyses, the present study shows the cellular programs that couple epithelial remodeling to immune activation in human gallbladder inflammation.

Building on our previous findings in mice that biliary epithelial cells act as immunocompetent effectors capable of releasing inflammatory mediators or directly modulating smooth-muscle tone via chemosensory-dependent and -independent mechanisms (Keshavarz et al. 2022, 2024; Ruppert et al. 2020), the current study investigates region-specific epithelial remodeling and immune-cell interactions in human gallbladder tissue from acute and chronic inflammatory conditions. Using ultrastructural analysis, immunohistochemistry, and single-cell transcriptomic profiling, we characterize epithelial integrity, mucin accumulation and impaired secretion, and immune infiltration across the neck, body, and peribiliary regions. Integrating regional morphological and molecular data, this work defines distinct epithelial and immune signatures associated with acute and chronic inflammation and provides new insights into how epithelial compartments and their immune microenvironments collectively shape the pathogenesis of human gallbladder disease.

## Methods

### Ethical approval

All procedures involving human participants were reviewed and approved by the Ethics Committee of Ludwig-Maximilians-Universität München (Project No. 23–0569) and by the relevant regulatory authorities in Munich, Germany. The study was performed in compliance with institutional and governmental regulations and the principles of the Declaration of Helsinki, with particular attention to the rights, privacy, and well-being of both patients and clinical personnel.

Eligible participants were adults (≥ 18 years) without selection based on age or sex. Exclusion criteria comprised chronic infectious diseases (e.g., HIV or hepatitis), known autoimmune or immunodeficiency disorders, current or previous secondary malignancies, and ongoing immunosuppressive or immunomodulatory treatment. Human tissue samples were obtained from patients undergoing routine cholecystectomy for cholelithiasis or cholecystitis.

Sample collection was limited to individuals who had provided written informed consent after receiving detailed oral and written information and sufficient time for consideration.

### Monitoring of clinical signs

The diagnosis of acute cholecystitis and chronic cholelithiasis was established based on clinical presentation, laboratory investigations, and imaging findings, in accordance with institutional diagnostic guidelines.

Upon hospital admission, all patients underwent a standardized laboratory workup, including complete blood count, C-reactive protein (CRP), liver function tests (alanine aminotransferase [ALT], aspartate aminotransferase [AST], alkaline phosphatase [AP], γ-glutamyltransferase [GGT]), and total and direct bilirubin levels. These parameters were used to evaluate inflammatory activity and hepatobiliary involvement.

Abdominal ultrasonography was routinely performed to confirm the presence of gallstones and to assess gallbladder wall thickening, pericholecystic fluid, and biliary dilatation. In selected cases, computed tomography (CT) or magnetic resonance cholangiopancreatography (MRCP) was additionally performed to further characterize the extent of inflammation and to exclude complications such as perforation or abscess formation.

Throughout hospitalization, all patients were clinically monitored according to institutional standards. Routine assessments included body temperature, heart rate, respiratory rate, blood pressure, and oxygen saturation. Special attention was given to abdominal findings including right upper quadrant tenderness, guarding, and Murphy’s sign and to the presence of nausea, vomiting, jaundice, or systemic infection. Pain intensity was evaluated using a standardized Numeric Rating Scale (NRS).

Serial laboratory tests (white blood cell count, CRP, liver enzymes, and bilirubin levels) and follow-up imaging were performed when clinically indicated to monitor disease progression and treatment response. All clinical and diagnostic data were recorded in the patients’ medical files and anonymized for study analysis in compliance with ethical and data protection regulations.

### Histopathological analysis

Gallbladder tissues from eight patients with acute cholecystitis and eight patients with chronic cholelithiasis were fixed in 10% neutral-buffered formalin for a minimum of 24 h to ensure optimal preservation, embedded in paraffin, and sectioned at a thickness of 4–5 µm using a rotary microtome for subsequent histological and immunohistochemical analyses.

For routine histopathology, sections were stained with hematoxylin and eosin (H&E) according to standard protocols at the Department of Pathology, Augsburg Hospital. Hematoxylin staining highlighted nuclei in blue to purple hues, allowing assessment of nuclear morphology, inflammatory cell infiltration, and epithelial organization, while eosin counterstained cytoplasmic and extracellular structures in pink to red, enabling evaluation of tissue architecture, edema, fibrosis, and necrosis. H&E-stained sections were examined by board-certified pathologists using light microscopy.

For immunohistochemistry (IHC), paraffin sections were deparaffinized in xylene, rehydrated through graded ethanol, and subjected to heat-induced epitope retrieval in either citrate buffer (pH 6.0) or Tris–EDTA buffer (pH 9.0), depending on antigen requirements. Endogenous peroxidase activity was quenched by 3% hydrogen peroxide. Sections were incubated with mouse monoclonal primary antibodies against CD163 (clone MRQ-26; ready-to-use; Roche, Basel, Switzerland), CD15 (clone MMA; 1:50), CD68 (clone KP-1; 1:100), cytokeratin 18 (CK18; clone DC10; 1:100), cytokeratin 19 (CK19; clone A53B/A2,26; 1:100), epithelial membrane antigen (EMA; clone E29; 1:200), MUC1 (clone MRQ-17; 1:50), and MUC4 (clone 8G7; ready-to-use). Except for CD163, all antibodies were obtained from Cell Marque (Rocklin, CA, USA) or BioSB (Santa Barbara, CA, USA). In addition, negative controls were included in each staining run by omitting the primary antibody on gallbladder sections as well as on control sections from other tissues (lymphoid tissue, prostate, colon, breast, and liver).

Antibody binding was visualized using an HRP-conjugated detection system (OptiView DAB IHC Detection Kit; Ventana Roche, Tucson, AZ, USA) with 3,3′-diaminobenzidine (DAB) as chromogen, followed by counterstaining with hematoxylin (DAB Hematoxylin and Bluing Reagent; Ventana Roche). Stained slides were dehydrated, cleared, and mounted for evaluation.

Microscopic analysis was performed using a bright-field microscope (Olympus BX43; Olympus Corporation, Tokyo, Japan). Immunoreactivity was evaluated qualitatively, focusing on the localization and distribution of staining within epithelial and subepithelial compartments. Observations included the presence or absence of marker expression and the overall staining pattern. Gross pathology images were obtained intraoperatively or immediately following tissue resection.

### RNA-Seq-based cell composition estimation and differential gene-expression profiling

To assess cellular composition and transcriptional differences, single-cell RNA sequencing (scRNA-seq) was performed on gallbladder tissue samples obtained from two patients, one with acute cholecystitis and one with chronic cholelithiasis. Tissue dissociation and single-cell suspension preparation were conducted according to established protocols using Singleron’s proprietary sCelLive Tissue Dissociation Buffer at the facilities of Singleron Biotechnologies GmbH (Cologne, Germany).

The resulting single-cell suspension was loaded onto SCOPE-chips to capture individual cells within microwells. Messenger RNA (mRNA) from each cell was barcoded using GEXSCOPE Single-Cell mRNA Library Kits (Singleron Biotechnologies), ensuring that transcripts from each cell received a unique molecular identifier. The captured mRNA was reversed transcribed into complementary DNA (cDNA), which was subsequently amplified and fragmented. Illumina-compatible sequencing libraries were then prepared by adapter ligation followed by PCR amplification.

The libraries were sequenced on an Illumina NovaSeq 6000 platform using an S4 flow cell with a paired-end 150-bp configuration, achieving an average sequencing depth of approximately 50,000 reads per cell. From the original dataset of two whole gallbladders suffering from acute and chronic inflammation, immune cells were extracted based on the expression of *Cd45* gene (*Ptprc* > 0). To extract only cells in “healthy state,” additional exclusion criteria were applied: (1) total number of genes expressed by cell was set to a minimum of 200 and a maximum of 7000 genes per cell (Fig. S5 a), (2) all the cells expressing more than 15% of mRNA were excluded (Fig. S5 b). The expression profiles of 8490 immune cells, 4458 from a patient with acute gallbladder inflammation and 4032 from a patient with chronic gallbladder inflammation, were analyzed on a Linux-based local server (Ubuntu v24.04, Intel i9-12900KF processor, 128 GB DDR4 RAM, Samsung 990 PRO M.2 NVMe 1 TB hard drive). Data analysis was performed in R (v4.4.0) using the following libraries: Seurat (v4.3.0), patchwork (v1.3.2), dplyr (v1.1.4), and clusterProfiler (v4.16.0) (Hao et al. 2024; Yu et al. 2012). The Louvain algorithm (resolution = 0.4) was applied for clustering, and the uniform manifold approximation and projection (UMAP; dimensions = 1–10) for visualization. A total of 12 clusters were identified across the two conditions.

### PCR analysis of epithelial marker expression

Gallbladder tissue samples from thirteen patients, five with acute cholecystitis and eight with chronic cholelithiasis, were collected from patients undergoing cholecystectomy due to acute cholecystitis or chronic cholelithiasis. Samples were immediately homogenized in RLT Plus lysis buffer (QIAGEN, 51,304) and stored at − 80 °C until processing.

Total RNA was extracted using the RNeasy Mini Kit (QIAGEN) following the manufacturer’s protocol. To remove residual genomic DNA, samples were treated with DNase I (Invitrogen) at 1 U per µg RNA. First-strand cDNA synthesis was performed at 42 °C for 50 min using Superscript II reverse transcriptase (200 U/µg RNA; Invitrogen, 18,064,014).

For conventional RT-PCR, 1 µl of cDNA was amplified using gene-specific primers for Epithelial cell adhesion molecule (EPCAM; F: GACTTTTGCCGCAGCTCAGGA; R: AGCAGTTTACGGCCAGCTTGT) and the reference gene β2-microglobulin (βMG; F: GTCTCGCTCCGTGGCCTTAG; R: TCAATGTCGGATGGATGAAACC). Each 25 µl reaction contained 0.3 µl of each primer, 2.5 µl of 10 × PCR buffer II (100 mM Tris–HCl, 500 mM KCl, pH 8.3), 2 µl of 15 mM MgCl₂, 0.6 µl of 10 mM dNTP mix, 0.15 µl of AmpliTaq Gold polymerase (5 U/µl; Applied Biosystems), and nuclease-free water. Cycling conditions were as follows: 94 °C for 5 min, followed by 40 cycles of 94 °C for 20 s, 60 °C for 20 s, and 73 °C for 20 s, with a final extension at 72 °C for 7 min. PCR products were separated on 2% TAE agarose gels, visualized under UV illumination, and compared to a 100-bp DNA ladder (Invitrogen, 15,628,050).

For quantitative expression analysis (qPCR), reactions were performed on an iCycler platform (Bio-Rad, Germany) using iTaq Universal SYBR Green Supermix (Bio-Rad Laboratories, CA, USA). Primer pairs for EPCAM and B2M were identical to those used in conventional PCR. Cycling conditions were 95 °C for 3 min, followed by 45 cycles of 95 °C for 15 s, 61 °C for 15 s, and 72 °C for 15 s. Relative gene-expression levels were calculated using the ΔCT method, with B2M as the internal reference gene.

### Electron microscopy and stereology

For transmission electron microscopy analysis, human gallbladder specimens from eighteen patients, nine with acute cholecystitis and nine with chronic cholelithiasis, were obtained from patients undergoing cholecystectomy. Tissues were rinsed in PBS and fixed in Zamboni solution for 24 h, then washed in 0.1 M Tris–HCl buffer, post-fixed in 1% osmium tetroxide (OsO₄) for 2 h, and rinsed with distilled water (5 × 5 min). Samples were stained en bloc overnight with 1% aqueous uranyl acetate, washed again (5 × 5 min), dehydrated through a graded ethanol series, and embedded in Agar 100 (Plano, Wetzlar, Germany, Cat# R1031).

Ultrathin sections (~ 80 nm) were cut using a Reichert Ultracut E ultramicrotome (Leica) and examined with a Zeiss EM 902 transmission electron microscope equipped with a 2 K CCD camera (TRS, Tröndle, Moorenweis, Germany).

Cholangiocytes were included for analysis only if they were sectioned along their full apicobasal axis—defined as having at least 12 µm of luminal contact and reaching the basal lamina. Based on the method of Baur (1969), a minimum of six cholangiocytes per specimen was determined to be sufficient to ensure that an additional outlier (± 3 SD from the mean) would alter the mean by no more than 10%. Accordingly, 6–10 cells were evaluated per sample. For ultrastructural quantification, transmission electron micrographs acquired at a final magnification of × 7000 were overlaid with a standardized stereological mesh grid, and mucin granule volume density was determined by point counting within the uppermost 1 µm of the apical cytoplasmic compartment of cholangiocytes, using an approach adapted from our previously published murine gallbladder studies (Keshavarz et al. 2022). Quantitative analysis was performed by an investigator blinded to disease group allocation.

### Statistical analysis

All statistical analyses were performed using GraphPad Prism 9 (GraphPad Software, San Diego, CA, USA). Data points (“n”) represent biological replicates, corresponding to independent human tissue samples. Experimental reproducibility was verified through independent repetitions, as described in the respective figure legends. Individual data points and mean ± SEM are shown in scatter plots to illustrate variability and reproducibility.

Data distribution was assessed using the Kolmogorov–Smirnov and Shapiro–Wilk tests to evaluate normality and variance assumptions. As most datasets did not follow a normal distribution, the non-parametric Mann–Whitney *U* test was applied to compare acute cholecystitis and chronic cholelithiasis groups. Sample sizes were not statistically predetermined, except for the stereological analysis of cholangiocytes, which was designed according to the Baur method (as described above). A *p*-value ≤ 0.05 was considered statistically significant.

## Results

### Clinical and biochemical profiles of acute and chronic gallbladder inflammation

A total of 41 patients were analyzed, including 12 with acute cholecystitis and 29 with chronic cholelithiasis. Classification was based on clinical presentation, laboratory findings, and imaging results. Abdominal ultrasound confirmed the presence of gallstones and assessed gallbladder wall thickening or pericholecystic fluid; in selected cases, CT or MRCP was performed in addition to evaluate disease severity. To assess systemic inflammation, routine blood parameters were compared between the two patient groups (Table 1). White blood cell (WBC) counts and C-reactive protein (CRP) levels were significantly elevated in acute cholecystitis (WBC = 12.41 ± 0.84 vs. 9.31 ± 0.62 × 10^9^/l, *p* < 0.0001; CRP = 19.64 ± 3.52 vs. 6.83 ± 1.90 mg/l, *p* = 0.0161). Total bilirubin and alkaline phosphatase (AP) were also higher in acute cases (total bilirubin = 0.78 ± 0.105 vs. 0.59 ± 0.068 mg/dl, *p* < 0.0001; AP = 135.18 ± 18.75 vs. 103.25 ± 9.90 U/l, *p* < 0.0001). Interestingly, alanine aminotransferase (ALT) levels were lower in acute compared to chronic cases (26.25 ± 5.40 vs. 42.68 ± 10.13 U/l, *p* = 0.0001), whereas aspartate aminotransferase (AST), gamma-glutamyl transferase (GGT), and direct bilirubin showed no significant differences between groups. Chronic cholelithiasis represented a more heterogeneous condition, encompassing cases with minimal inflammatory activity as well as long-standing fibrotic or epithelial remodeling changes. Accordingly, systemic inflammatory markers in this group were generally low or within normal limits, contrasting with the pronounced acute-phase response observed in acute cholecystitis.

**Table 1:** Clinical and biochemical parameters of patients with acute cholecystitis and chronic cholelithiasis. Comparison of systemic inflammatory and hepatic function markers between patients diagnosed with acute cholecystitis and those with chronic cholelithiasis.

Parameter	Acute cholecystitis Mean ± SEM	Chronic cholelithiasis Mean ± SEM	*p*-value
White blood cells (WBC)	12.409 ± 0.84, *n* = 12	9.31 ± 0.62, *n* = 29	< 0.0001
C-reactive protein (CRP)	19.64 ± 3.52, *n* = 12	6.83 ± 1.90, *n* = 28	0.0161
Alanine aminotransferase (ALT)	26.25 ± 5.40, *n* = 12	42.68 ± 10.13, *n* = 29	0.0001
Aspartate aminotransferase (AST)	36.90 ± 4.21, *n* = 12	39.91 ± 3.56, *n* = 24	0.2692
Alkaline phosphatase (AP)	135.18 ± 18.75, *n* = 12	103.25 ± 9.90, *n* = 28	< 0.0001
Gamma-glutamyl transferase (GGT)	102.75 ± 27.09, *n* = 12	129.51 ± 32.78, *n* = 29	0.3612
Total bilirubin	0.78 ± 0.105, *n* = 12	0.59 ± 0.068, *n* = 29	< 0.0001
Direct bilirubin	0.48 ± 0.057, *n* = 12	0.37 ± 0.037, *n* = 29	0.2751

Acute cholecystitis was associated with significantly elevated white blood cell (WBC) counts, C-reactive protein (CRP), alkaline phosphatase (AP), alanine aminotransferase (ALT), and total bilirubin levels compared to the chronic group, indicating acute inflammatory and cholestatic responses. Data are presented as mean ± SEM; statistical significance was determined using the Mann–Whitney test.

### Histopathological characteristics of acute and chronic gallbladder inflammation

**Fig. 1:**
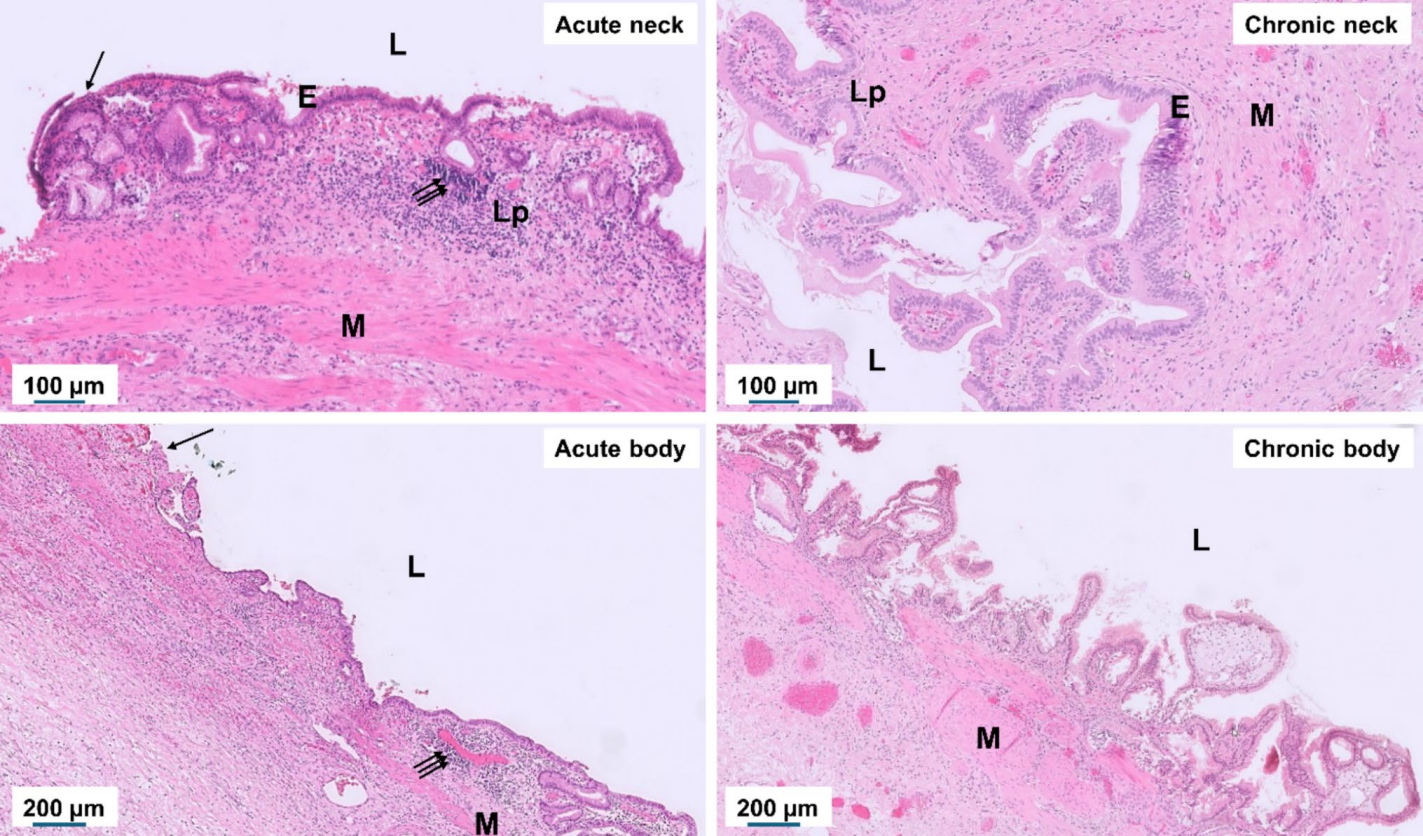
Histopathological features of acute cholecystitis and chronic cholelithiasis in the gallbladder neck and body. Representative hematoxylin and eosin (H&E)–stained sections illustrating characteristic epithelial and stromal alterations under acute and chronic conditions. The acute (cholecystitis) neck and body show epithelial disruption (single arrow), mucosal erosion, edema, and dense neutrophilic infiltration (triple arrows). In contrast, the chronic (systematic cholelithiasis) neck and body display preserved but hyperplastic epithelium with papillary projections, stromal fibrosis, and glandular proliferation, indicative of long-standing tissue remodeling. L, lumen; E, epithelium; Lp, lamina propria; M, muscle layer. Scale bars: 100 µm (neck), 200 µm (body).

Histological evaluation was performed on eight acute cholecystitis and eight chronic cholelithiasis specimens, revealing distinct morphological patterns between the two conditions (Fig. 1). In acute cholecystitis, the gallbladder wall exhibited extensive neutrophilic and macrophage infiltration, most prominently in the neck region, accompanied by epithelial disruption, mucosal erosion, interstitial edema, and focal necrosis (triple arrows). The inflammatory process often extended through the muscular layer, reflecting an active and destructive phase of inflammation. In contrast, chronic cholelithiasis specimens displayed preserved epithelial continuity but with pronounced epithelial hyperplasia and papillary folding. The subepithelial stroma showed dense collagen deposition and marked fibrosis, with minimal residual inflammatory cells. In the chronic body region, the mucosal epithelium formed deep invaginations extending into the muscular layer (Rokitansky–Aschoff sinuses), accompanied by perimuscular fibrosis, indicating long-standing structural remodeling rather than ongoing inflammation. Overall, these findings demonstrate that acute cholecystitis is characterized by active inflammatory cell infiltration and tissue injury, whereas chronic cholelithiasis represents fibrotic wall thickening and epithelial remodeling following repeated or subclinical inflammatory episodes.

### Immunohistochemical characterization of regional inflammatory and epithelial responses

**Fig. 2:**
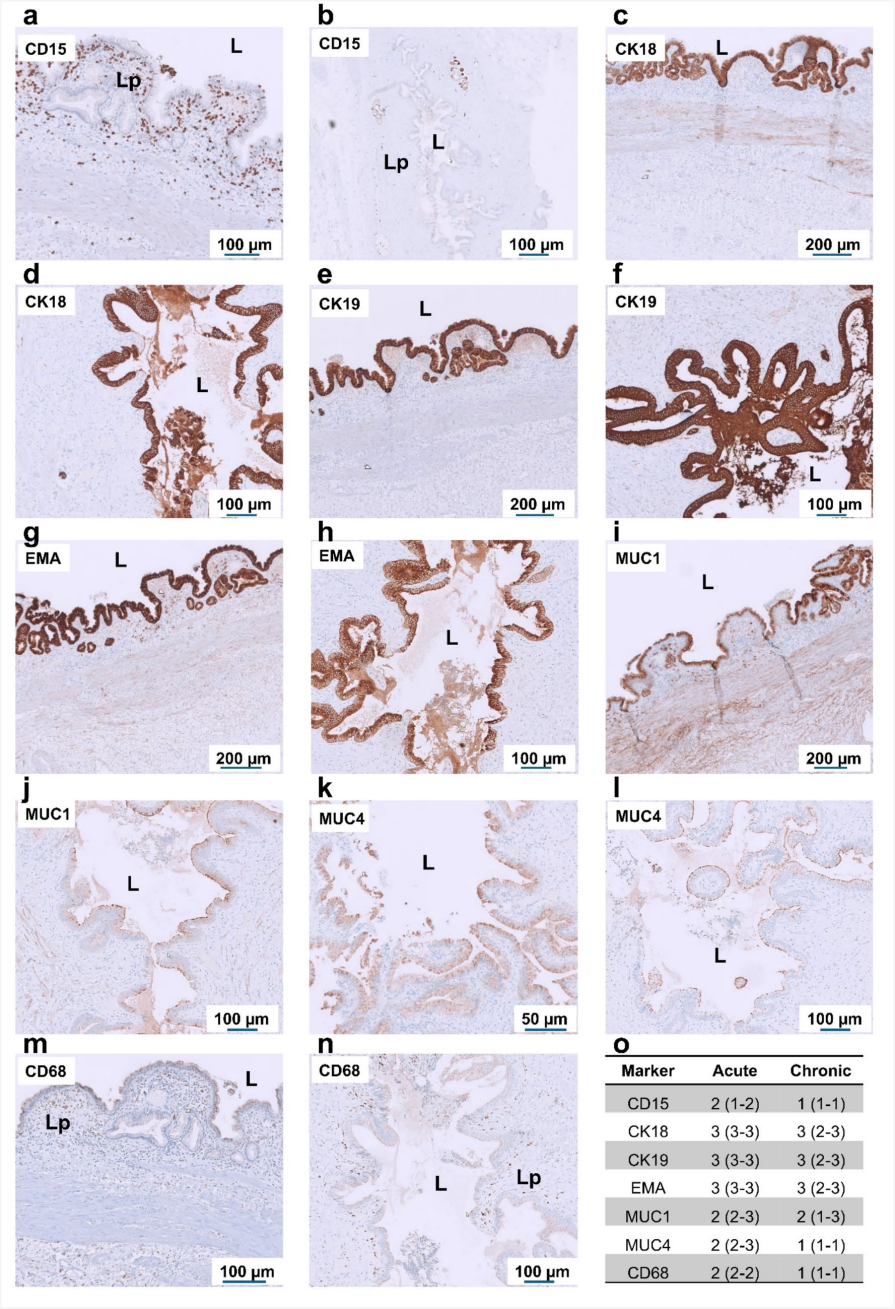
Immunohistochemical characterization of inflammatory and epithelial markers in the gallbladder neck during acute cholecystitis and chronic cholelithiasis. Representative immunostaining of gallbladder neck tissue from acute (**a**, **c**, **e**, **g**, **i**, **k**, **m**) and chronic (b, d, f, h, j, l, n) conditions, shown from left to right. Staining includes inflammatory marker CD15, epithelial cytokeratins CK18 and CK19, epithelial differentiation markers EMA, MUC1, and MUC4, followed by the macrophage marker CD68. In acute cholecystitis, pronounced epithelial and subepithelial staining for CD15, CK18, and CK19 reflects intense neutrophilic infiltration and epithelial activation, while CD68 highlights abundant macrophages within the inflamed lamina propria. In chronic cholelithiasis, inflammatory marker expression is reduced, whereas the epithelium remains preserved and exhibits heterogeneous MUC1 and MUC4 expression, consistent with epithelial remodeling. **o** Quantification of immunopositive cells and epithelial marker expression in the lamina propria and epithelial compartments of acute and chronic gallbladder samples (*n* = 4 per group). Data are presented as median with interquartile range (IQR). L, lumen; Lp, lamina propria. Scale bars: 50–200 µm (as indicated). Corresponding negative control stainings, including gallbladder sections and tissue-specific controls processed in parallel with omission of the primary antibody, are shown in Supplementary Figs. 1–4

**Fig. 3:**
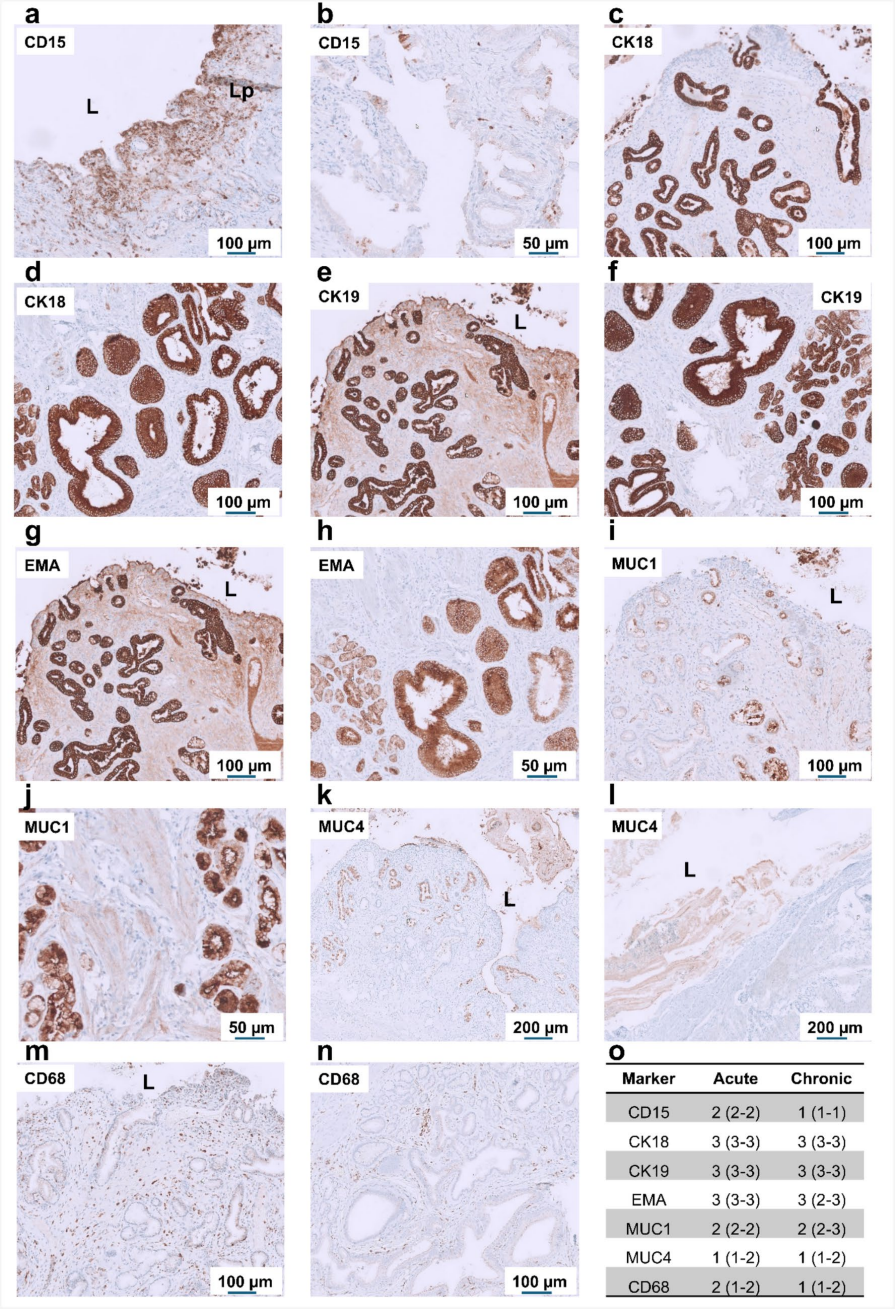
Immunohistochemical characterization of inflammatory and epithelial markers in the peribiliary glandular region during acute cholecystitis and chronic cholelithiasis. Representative immunostaining of peribiliary glandular regions from acute (**a**, **c**, **e**, **g**, **i**, **k**, **m**) and chronic (**b**, **d**, **f**, **h**, **j**, **l**, **n**) conditions, shown from left to right. Expression of inflammatory markers CD15 and CD68, epithelial cytokeratins CK18 and CK19, and epithelial differentiation markers EMA, MUC1, and MUC4 is depicted. In acute cholecystitis, intense epithelial and periductal staining for CD15, CK18, and CK19 indicates active neutrophil infiltration and epithelial activation, while CD68 highlights macrophages within the periductal lamina propria. In the acute state, epithelial and glandular cells display strong luminal and cytoplasmic expression of EMA, MUC1, and MUC4, reflecting glandular activation and mucin hypersecretion. In chronic cholelithiasis, inflammatory marker expression is reduced, whereas epithelial remodeling with heterogeneous MUC1 and MUC4 distribution indicates long-standing fibrotic transformation and tissue adaptation. **o** Quantification of immunopositive cells and epithelial marker expression in the peribiliary lamina propria and epithelial compartments of acute and chronic gallbladder samples (*n* = 4 per group). Data are presented as median with interquartile range (IQR). L, lumen; Lp, lamina propria. Scale bars: 50–200 µm (as indicated).

**Fig. 4:**
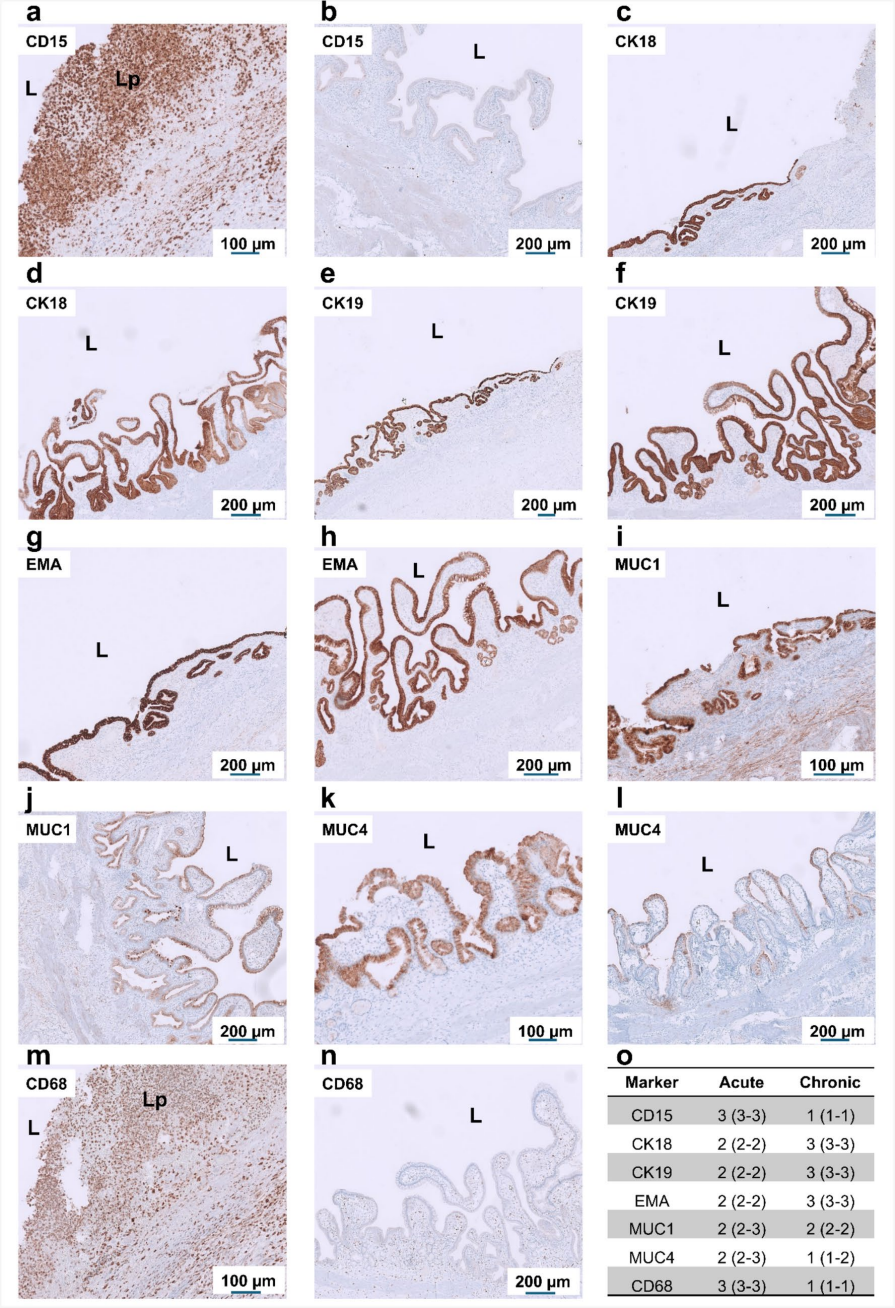
Immunohistochemical characterization of inflammatory and epithelial markers in the gallbladder body during acute cholecystitis and chronic cholelithiasis. Representative immunostaining of gallbladder body sections from acute (**a**, **c**, **e**, **g**, **i**, **k**, **m**) and chronic (**b**, **d**, **f**, **h**, **j**, **l**, **n**) conditions, shown from left to right. Staining includes epithelial cytokeratins CK18 and CK19, inflammatory marker CD15, epithelial differentiation markers EMA, MUC1, and MUC4, followed by the macrophage marker CD68. In acute cholecystitis, strong epithelial and subepithelial staining for CD15, CK18, and CK19 reflects dense neutrophil infiltration and epithelial activation, while CD68 highlights abundant macrophages within the inflamed lamina propria and stromal compartments. In the acute state, epithelial folds show continuous and intense luminal and cytoplasmic expression of EMA, MUC1, and MUC4, reflecting preserved epithelial integrity and increased secretory activity. In chronic cholelithiasis, inflammatory marker expression is reduced, whereas epithelial remodeling with variable MUC1 and MUC4 expression and diminished glandular activity indicates long-standing mucosal adaptation. **o** Quantification of immunopositive cells and epithelial marker expression in the lamina propria and epithelial compartments of acute and chronic samples (*n* = 4 per group). Data are presented as median with interquartile range (IQR). L, lumen; Lp, lamina propria. Scale bars: 100–200 µm (as indicated)

**Fig. 5:**
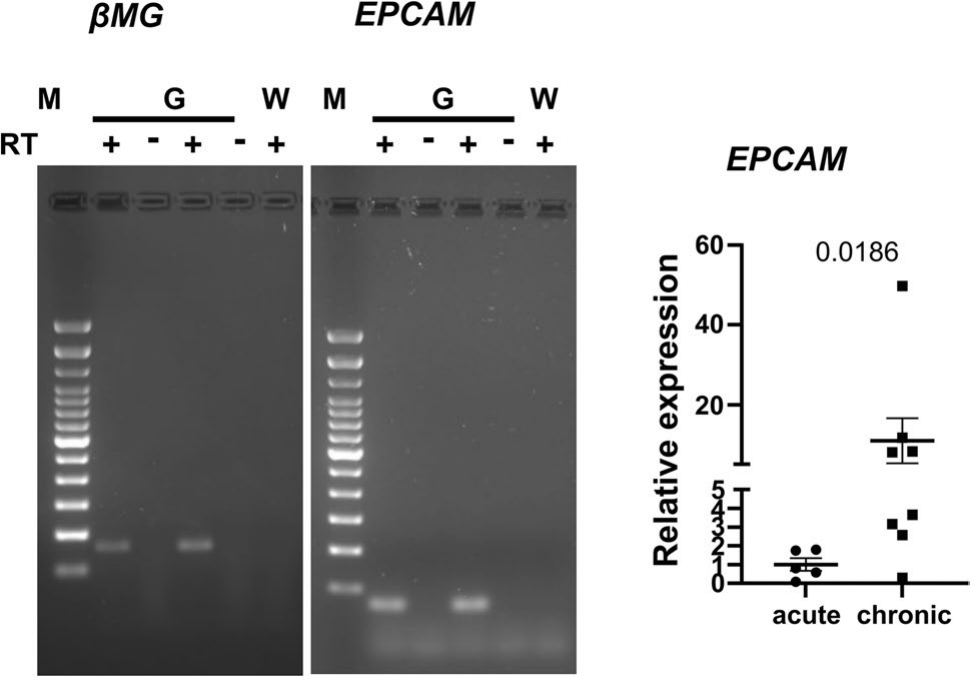
Reduced epithelial marker expression in acute cholecystitis compared with chronic cholelithiasis. RT-PCR and quantitative PCR analyses were performed to validate histological findings of epithelial abrasion in acute gallbladder tissue. Representative RT-PCR gel images show amplification of β2-microglobulin (βMG) as a housekeeping control and EPCAM in gallbladder (G) and water (W) as control, with (+) and without (−) reverse transcriptase (RT). Quantitative PCR confirmed a significant reduction in EPCAM expression in acute cholecystitis compared with chronic

Immunohistochemical analysis revealed region-specific differences in immune-cell infiltration and epithelial marker expression between acute cholecystitis and chronic cholelithiasis (Figs. 2, 3 and 4). Immune infiltration was assessed using CD15 (neutrophils) and CD68 (macrophages), while epithelial differentiation and mucin-associated remodeling were evaluated with EMA, MUC1, MUC4, CK18, and CK19. In the neck region (Fig. 2), acute cholecystitis was characterized by dense CD15^+^ neutrophil infiltration and scattered CD68^+^ macrophages, particularly beneath the mucosal surface. The epithelium exhibited strong and continuous expressions of EMA, MUC1, and MUC4, together with robust CK18 and CK19 labeling, indicating epithelial regeneration and mucin secretion. In contrast, chronic cholelithiasis specimens from the same region displayed markedly reduced immune staining, with only sparse CD15^+^ and CD68^+^ cells. The epithelial layer maintained strong expression of EMA, CK18, and CK19, confirming epithelial preservation and biliary-type differentiation, while MUC1 and MUC4 staining appeared weaker and more heterogeneous, consistent with altered mucin composition and fibrosis-associated remodeling. In the peribiliary region (Fig. 3), inflammatory cell density was generally lower than in the neck. Chronic specimens showed expanded EMA^+^/CK19^+^ duct-like invaginations and glandular structures, reflecting adaptive secretory remodeling and partial ductular transformation within the fibrotic stroma. In the body region (Fig. 4), acute samples exhibited focal subepithelial infiltration of CD15^+^ and CD68^+^ cells, while chronic cases contained few immune cells but pronounced stromal fibrosis. Epithelial markers (EMA, CK18, CK19, MUC1, MUC4) remained detectable; however, MUC1 and MUC4 expressions appeared weaker and discontinuous compared with acute inflammation. Collectively, these regional patterns demonstrate that acute cholecystitis shows intense inflammatory infiltration with reactive epithelial injury, while chronic cholelithiasis is characterized by fibrosis and preserved but remodeled epithelium. To further support the immunohistochemical findings, EPCAM expression, indicative of epithelial differentiation, was examined by RT-PCR and quantitative PCR using tissue from the gallbladder body region (Fig. 5). EPCAM mRNA was detected in both acute and chronic gallbladder tissues, with stronger signal intensity in chronic samples. Quantitative analysis confirmed significantly higher EPCAM expression in chronic cholelithiasis compared with acute cholecystitis (*p* = 0.0186), supporting the maintained epithelial differentiation and structural remodeling observed histologically.

### Ultrastructural features of epithelial remodeling in acute and chronic inflammation

**Fig. 6:**
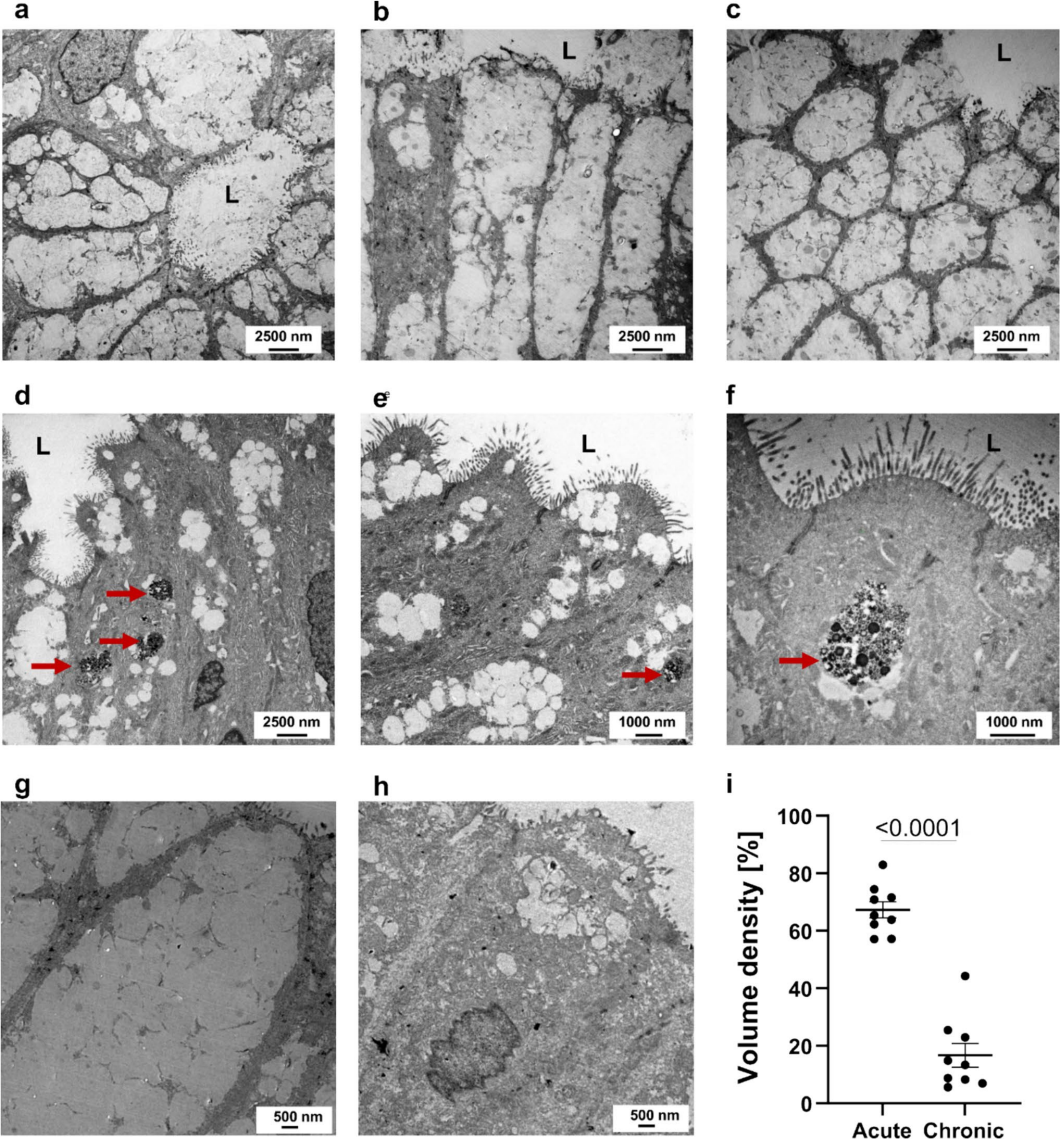
Ultrastructural alterations of the gallbladder epithelium in acute cholecystitis and chronic cholelithiasis. Transmission electron microscopy images showing epithelial morphology in acute (**a–c**) and chronic (**d–f**) conditions. In acute cholecystitis, epithelial cells display pronounced cytoplasmic vacuolization and numerous mucin granules. The luminal surface (L) retains microvilli, although some appear irregular or shortened. In chronic cholelithiasis, epithelial polarity and junctional integrity are preserved, and the cytoplasm contains electron-dense bodies and lysosomal inclusions (red arrows). **g**, **h** High-magnification views illustrate mucin granule–rich cytoplasm in acute tissue (**h**) and compact epithelial organization in chronic tissue (**g**). **i** Quantitative stereological analysis of epithelial volume density shows higher value in acute cholecystitis specimens. Scale bars are indicated in the panels

Transmission electron microscopy of epithelial tissue from the gallbladder body region revealed distinct ultrastructural differences between acute cholecystitis and chronic cholelithiasis (Fig. 6). In acute cases, epithelial cells contained numerous mucin-filled secretory vesicles, well-developed microvilli, and intact intercellular junctions, consistent with active mucin secretion and epithelial regeneration (Fig. 6a–c). The luminal surface displayed abundant glycocalyx-coated microvilli and exocytotic vesicles projecting into the lumen (L), indicating vigorous secretory activity and barrier maintenance. In chronic cholelithiasis, epithelial cells exhibited reduced mucin content, accumulation of electron-dense cytoplasmic bodies, and irregular apical membranes. These dense bodies varied in size and electron density and frequently corresponded to lipolysosomes or multivesicular bodies (MVBs), organelles associated with impaired degradation, chronic immune activation, and metabolic stress (Fig. 6d–f). MVB-like structures containing intraluminal vesicles are typically involved in exosome formation and cytokine signaling, whereas lipolysosomes represent specialized lysosomes that degrade lipid-rich material, reflecting chronic metabolic and oxidative adaptation. High-magnification images further confirmed the abundance of mucin granules in acute inflammation and dense bodies in chronic disease, indicating a shift from secretory activity toward degradative and metabolic remodeling of the epithelium (Fig. 6g–i). The ultrastructural volume density of mucin granules, an established stereological parameter reflecting epithelial glycoprotein secretion, was markedly reduced in chronic cholelithiasis compared with acute cholecystitis, supporting a decline in secretory activity and the predominance of degradative remodeling.

### Single-cell transcriptomic profiling of immune and stromal compartments

**Fig. 7:**
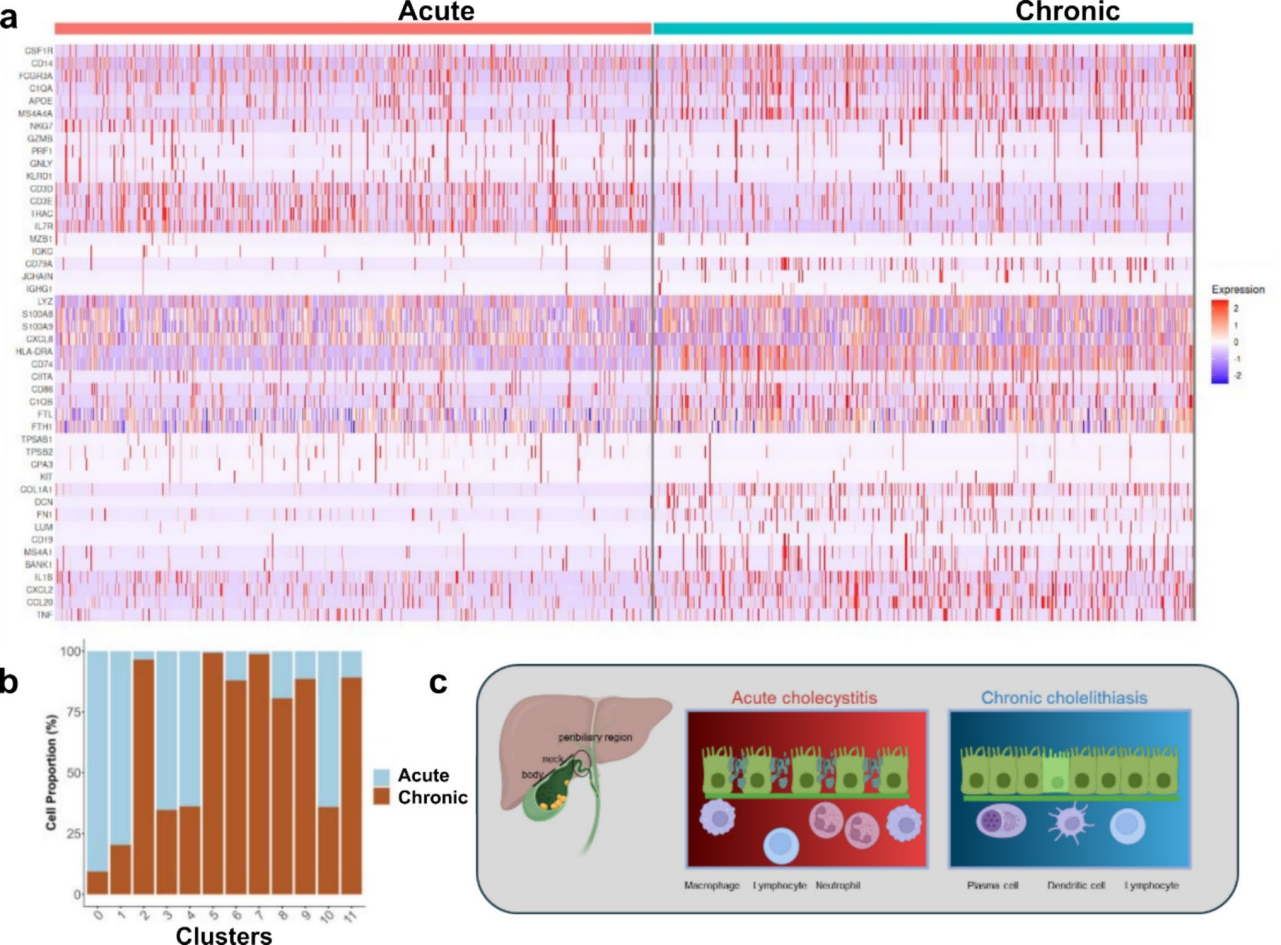
Transcriptomic and anatomical overview of immune and epithelial alterations in acute cholecystitis and chronic cholelithiasis. Single-cell RNA sequencing (scRNA-seq) of immune cells from one acute cholecystitis and one chronic cholelithiasis sample reveals distinct expression signatures and immune-cell distributions. **a** Heatmap describing the relative cell expression profiles in acute and chronic conditions based on inflammation- and fibrosis-associated marker genes, illustrating clear transcriptional differences between the two states. **b** Bar graph summarizing the relative abundance of major immune-cell populations across both conditions. Neutrophils (cluster 0), CD4^+^ T cells (clusters 1), macrophages/monocytes (clusters 3), cytotoxic lymphocytes (cluster 4), and mast cells (cluster 10) are more abundant in acute cholecystitis, whereas B/plasma cells (cluster 6), CXCL13^+^ epithelial-interacting macrophages (cluster 7), antigen-presenting macrophages/dendritic cells (cluster 8), fibroblasts/myofibroblasts (cluster 9), and cycling immune cells (cluster 11) predominate in chronic cholelithiasis. **c** Schematic representation of the analyzed gallbladder regions (body, neck, and peribiliary area) and corresponding epithelial and immune features—acute cholecystitis showing epithelial disruption and infiltration by innate immune cells, and chronic cholelithiasis displaying preserved or hyperplastic epithelium with adaptive-cell predominance, stromal remodeling

To complement the histological, immunohistochemical, and ultrastructural analyses, single-cell RNA sequencing (scRNA-seq) was performed on immune-enriched cell suspensions isolated from combined neck and body regions of one representative acute cholecystitis specimen and one chronic cholelithiasis specimen (Fig. 7; Figs. S5–S8). Although based on one sample per condition, the dataset provides an informative transcriptomic snapshot of immune and stromal cell states underlying acute and chronic gallbladder inflammation.

Quality-control analysis confirmed high sequencing integrity and comparable library complexity between the two samples (Fig. S5). Violin and scatter plots of nFeature_RNA, nCount_RNA, and percent.mt demonstrated consistent gene and transcript detection per cell and low mitochondrial RNA content, confirming cell viability and uniform sequencing depth across clusters (Fig. S5). Following quality assessment, cell clustering and annotation were performed using canonical marker genes (Fig. S6). To visualize transcriptional relationships within each cluster, gene-expression patterns were displayed as heatmaps and UMAP feature plots generated with Seurat’s visualization tools. This allowed comparative assessment of expression profiles between acute and chronic conditions across all major immune and stromal cell populations.

To visualize transcriptional patterns within each identified cluster, gene-expression data were displayed as heatmaps and UMAP feature plots generated with Seurat’s built-in plotting functions. Distinct clustering patterns separated acute and chronic samples, reflecting condition-specific immune activation states. In acute cholecystitis, neutrophil/granulocyte (cluster 0), CD4^+^ T-cell (cluster 1), and macrophage/monocyte populations (cluster 3) predominated, showing elevated expression of pro-inflammatory genes including *CSF1R*, *CD14*, *FCGR3A*, *S100A8*, and *CXCL8*. Conversely, chronic cholelithiasis was enriched for B/plasma-cell lineages (cluster 6), CXCL13^+^ epithelial-interacting macrophages (cluster 7), antigen-presenting macrophages/dendritic cells (cluster 8), and fibroblast/myofibroblast populations (cluster 9) expressing fibrosis- and matrix-associated genes such as *COL1A1*, *DCN*, *FN1*, and *LUM* (Fig. S7).

To further refine these observations, cell type–specific differential expression was analyzed across the immune compartment. Acute inflammation showed marked upregulation of *CSF1R*, *CD14*, *CXCL8*, *S100A8/A9*, and cytotoxic mediators (*GZMB*, *PRF1*, *GNLY*), indicating an activated myeloid and cytotoxic T/NK phenotype. In contrast, chronic cholelithiasis exhibited enhanced expression of B-cell and plasma-cell markers (*MZB1*, *IGKC*, *JCHAIN*, *CD79A*), antigen-presentation molecules (*HLA-DRA*, *CD74*, *CIITA*), and fibrotic-matrix genes (*COL1A1*, *FN1*, *DCN*, *LUM*), consistent with long-term tissue remodeling and adaptive immune predominance. Differential gene-expression and gene-set enrichment analyses revealed transcriptional programs paralleling the histopathological observations (Figs. S7–S8a).

Acute inflammation was associated with activation of mitochondrial oxidative stress, metabolic, and cytokine response pathways, whereas chronic cholelithiasis exhibited upregulation of immunoglobulin A (IgA)–related and secretory immune functions, consistent with transition toward immune adaptation and fibrotic tissue remodeling (Fig. S8b). Gene ontology enrichment further indicated that acute immune cells were metabolically active and energy-dependent, while chronic immune cells favored antibody production and extracellular matrix organization, suggesting a metabolic-to-structural transition in the inflammatory environment. When integrated with the morphological and ultrastructural findings (Fig. 7), the single-cell transcriptomic data highlight coordinated immune remodeling and its correspondence with epithelial and stromal changes observed in tissue analyses. Acute cholecystitis exhibits a neutrophil- and macrophage-dominated exudative profile with strong pro-inflammatory and metabolic activation, corresponding to the epithelial injury and mucin secretion seen histologically. In contrast, chronic cholelithiasis is characterized by lymphoid and stromal enrichment, fibrosis-related gene expression, and preservation or hyperplasia of the epithelium, in agreement with the adaptive and fibrotic remodeling demonstrated by histology and electron microscopy.

Together, these transcriptomic data delineate a molecular continuum from acute, cytokine-driven, and metabolically active inflammation to chronic, lymphoplasmacytic remodeling, and fibrotic transformation of the gallbladder mucosa.

### Targeted molecular validation of immune and stromal compartments

**Fig. 8:**
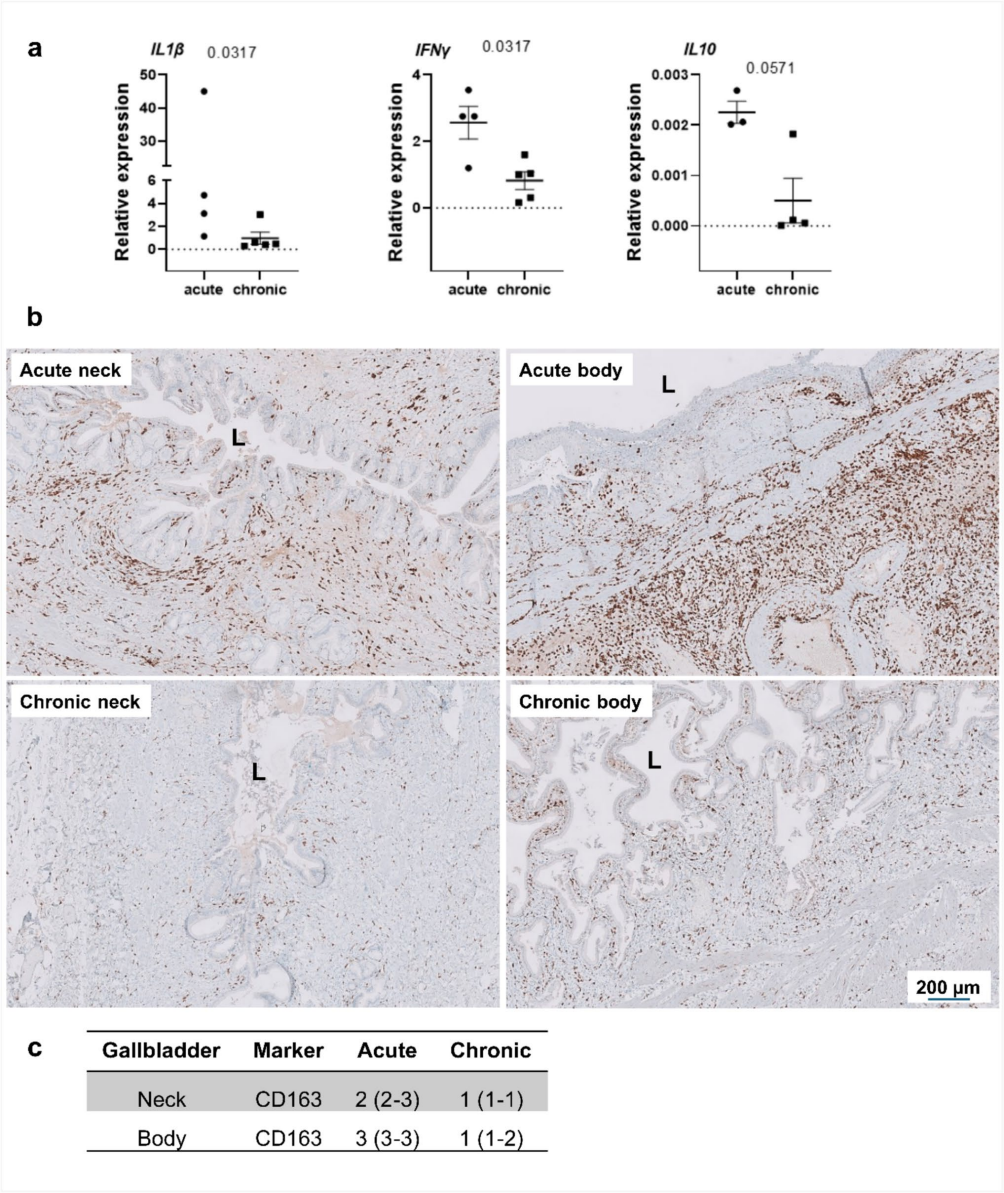
Targeted validation of immune activation states in acute cholecystitis and chronic cholelithiasis. Targeted gene-expression and immunohistochemical analyses were performed to validate immune signatures identified by single-cell transcriptomic profiling. **a** Quantitative PCR analysis of inflammatory cytokine expression in independent gallbladder tissue samples demonstrates significantly increased expression of IL1B and IFNG in acute cholecystitis compared with chronic cholelithiasis, whereas IL10 shows a trend toward increased expression in acute inflammation. Data are shown as individual values with median and interquartile range; statistical significance was assessed using the Mann–Whitney *U* test. **b** Representative immunohistochemical staining for CD163 in the gallbladder neck and peribiliary regions from acute and chronic conditions. Acute cholecystitis shows increased abundance of CD163^+^ macrophages within the mucosa and subepithelial compartments compared with chronic cholelithiasis. **c** Quantification of CD163.^+^ cells in the gallbladder neck and body (*n* = 4 per group) demonstrates a significantly higher density in acute inflammation. Scale bars: 200 µm (as indicated). Negative control stainings for CD163 are shown in Supplementary Figs. 1 and 2

To validate key immune signatures identified by single-cell transcriptomics, we performed targeted gene-expression and immunohistochemical analyses in independent gallbladder samples (Fig. 8). qPCR analysis demonstrated significantly elevated IL1B and IFNG expression in acute cholecystitis, whereas IL10 showed a trend toward increased expression, consistent with a predominantly innate inflammatory response accompanied by early counter-regulatory signaling. Immunohistochemical analysis revealed increased abundance of CD163^+^ macrophages in acute tissue compared with chronic cholelithiasis, supporting the presence of macrophage populations associated with tissue stress and resolution. Together, these findings provide complementary validation of the immune states inferred from scRNA-seq analysis.

## Discussion

The gallbladder is increasingly recognized as an immune-responsive epithelial organ that not only stores and concentrates bile but also contributes to mucosal defense and immune surveillance. Under physiological conditions, its epithelium maintains tight junction integrity, low proliferative activity, and a stable mucin barrier. Upon obstruction or infection, however, the tissue rapidly adopts a reactive phenotype characterized by cytokine release, immune-cell infiltration, and remodeling of the epithelial–stromal interface (Lan et al. 2022; Wilkins et al. 2017). These processes are not confined to the gallbladder: chronic inflammation of the biliary tree promotes liver and extrahepatic tissue damage and represents a leading cause of liver transplantation (Fabris et al. 2017; Jansen et al. 2017; Jia et al. 2018). Despite its clinical relevance, biliary inflammation remains poorly characterized at the cellular and molecular levels. Understanding epithelial–immune interactions in the extrahepatic biliary system is therefore central to elucidating common cholestatic and inflammatory disorders.

Our combined histological, ultrastructural, and single-cell transcriptomic analyses delineate this transformation as a continuum rather than a dichotomy, revealing coordinated immune and stromal remodeling from acute cholecystitis to chronic cholelithiasis. Acute cholecystitis typically arises from transient cystic-duct obstruction and bacterial colonization, most frequently by *Escherichia coli*, *Klebsiella*, or *Enterococcus*, that activate the innate immune system through endotoxin-driven signaling (Indar and Beckingham 2002; Lam et al. 2021). Elevated intraductal pressure and ischemia compromise epithelial integrity and permit translocation of bacterial components, triggering local and systemic cytokine cascades (Mosler 2011). Radiologic manifestations such as mural enhancement and pericholecystic fluid (Catalano et al. 2009) correspond to the vascular congestion and edema observed histologically. In our acute samples, neutrophils and macrophages dominated the infiltrate, while epithelial MUC1 and MUC4 expressions reflected an activated secretory state. Single-cell transcriptomics confirmed upregulation of macrophage and cytotoxic programs (CSF1R, CD14, FCGR3A, C1QA, NKG7, GZMB, PRF1) together with pro-inflammatory mediators (S100A8/A9, CXCL8, TNF), mirroring the innate activation described in acute bacterial cholangitis (Tong and Lou 2025; Wu et al. 2011; Yang Zhou 2023). These findings are consistent with the murine study by O’Leary et al., which identified a specialized biliary epithelial subpopulation acting as a bile acid–sensitive regulator of inflammation; loss of this cell type provoked spontaneous neutrophil infiltration and an epithelial inflammatory signature (O'Leary et al. 2022). In our human samples, ultrastructural examination revealed epithelial swelling, disrupted junctions, and microvillar shortening, features indicative of increased permeability and secretory exhaustion during the acute inflammatory burst.

In contrast, chronic cholelithiasis represents a remodeling phase characterized by reduced systemic inflammation but persistent local immune activation. Dense lymphoplasmacytic infiltrates, peribiliary-gland hyperplasia, and fibrosis, most prominent in the neck region, indicate a shift in immune organization toward adaptive immunity and stromal activation. Single-cell transcriptomic profiles revealed enrichment of B-lineage and plasma-cell markers (MZB1, IGKC, JCHAIN, CD79A, CIITA) and fibroblast-associated genes (COL1A1, FN1, LUM, DCN), reflecting matrix deposition and fibroblast reprogramming. Such immune-regulated biliary repair mechanisms, orchestrated by macrophages and lymphocytes, parallel those described in hepatic and intestinal fibrosis (Lan et al. 2022; Locatelli et al. 2016; Yao and Tang 2022).

In chronic inflammation, we identified macrophage subsets consistent with anti-inflammatory and epithelial-interacting phenotypes (clusters 5 and 7), suggestive of tissue adaptation rather than acute immune activation. Such macrophages are known to secrete trophic and matrix-modulating factors that may facilitate epithelial remodeling and fibrosis. Comparable macrophage polarization toward integrin αvβ6-inducing and MMP-modulating states has been implicated in biliary epithelial restitution following injury (Guillot et al. 2021). These mechanisms could underlie the ultrastructural alterations observed in chronic tissue depletion of mucin granules and accumulation of dense bodies and multivesicular vesicles indicative of metabolic stress and prolonged immune–epithelial interaction.

The depletion and abnormal storage of mucin observed in acute inflammation suggest that distinct epithelial subpopulations may modulate mucin release and immune signaling. Previous experimental work in mice showed that disruption of epithelial chemosensory signaling impairs mucin secretion and alters epithelial–immune communication in the gallbladder and extrahepatic biliary tract (Keshavarz et al. 2022). Specialized biliary epithelial cells have been proposed to sense luminal cues and transduce them via mediators such as acetylcholine, leukotrienes, and prostaglandins (Howitt et al. 2016; Nadjsombati et al. 2018; Perniss et al. 2023). Consistent with this concept, O’Leary et al. reported that bile acids regulate chemosensory epithelial abundance in mice and that loss of these cells enhances neutrophil infiltration and biliary inflammation (O'Leary et al. 2022). Considering these findings, our human data suggest that epithelial stress responses and local immune activation are tightly linked across disease stages.

Building on this, our present single-cell and ultrastructural analyses demonstrate that epithelial–immune interactions persist throughout both acute and chronic inflammation. Epithelial stress signatures coincided with macrophage expression of cytokines (IL1B, TNF) and trophic mediators (AREG, TGFB1), indicating reciprocal regulation of epithelial repair and immune activation. These feedback loops likely determine whether inflammation resolves, stabilizes, or progresses toward fibrosis and dysplasia. Comparable epithelial–immune circuits have been described in intestinal barrier repair and hepatic fibrosis (Kurashima et al. 2019; Wynn and Vannella 2016), underscoring conserved mucosal repair and immune regulatory frameworks. Consistent with these transcriptomic observations, targeted validation in independent tissue samples confirmed key immune activation signatures. Quantitative PCR analysis demonstrated significantly increased IL1B and IFNG expression in acute cholecystitis, while IL10 showed a trend toward increased expression, indicating dominant innate immune activation accompanied by early counter-regulatory signaling. Immunohistochemical analysis further revealed an increased abundance of CD163^+^ macrophages in acute tissue, supporting the presence of macrophage populations associated with tissue stress and resolution. Together, these findings corroborate the coexistence of pro-inflammatory and reparative immune programs during acute gallbladder inflammation.

In conclusion, this study defines the gallbladder as an immunologically active epithelial organ in which structural remodeling and immune activation are tightly interconnected. Persistent immune–epithelial crosstalk and bile acid–mediated signaling could, over time, create a microenvironment that favors epithelial plasticity and potentially neoplastic transformation. Recent reviews emphasize that bile acids and microbiota-derived metabolites modulate both innate and adaptive immune responses through receptors such as FXR, TGR5, and VDR, influencing tissue inflammation and the tumor immune microenvironment (Tong and Lou 2025). These insights underscore the importance of integrating immunometabolic and microbiome pathways into future studies of biliary inflammation and carcinogenesis. Although our analyses primarily focused on immune and stromal compartments, the combined histological, ultrastructural, and single-cell data demonstrate that the epithelium actively shapes local inflammatory responses rather than serving as a passive target. The acute phase of cholecystitis is dominated by cytokine-driven recruitment of neutrophils and macrophages, whereas chronic inflammation involves sustained adaptive immune and fibroblast activation through macrophage–B-cell–fibroblast networks. Future work should dissect how epithelial stress signaling and macrophage polarization jointly govern mucin release, epithelial plasticity, and fibrotic remodeling in human gallbladder disease.

## Data Availability

The RNA sequencing data generated and analyzed in this study are part of an ongoing research project and are not publicly available at this stage due to confidentiality agreements and data-protection considerations. Data can be shared by the corresponding author, Dr. Maryam Keshavarz, upon request. Competing Interest declaration: The authors declare no competing interests.
